# Statut phospho-calcique en hémodialyse chronique dans l’Oriental Marocain: évaluation de l’adhésion aux recommandations K/DOQI et KDIGO

**DOI:** 10.11604/pamj.2013.16.23.1959

**Published:** 2013-09-19

**Authors:** Nawal Benabdellah, Ilham Karimi, Yassamine Bentata, Hicham Yacoubi, Intissar Haddiya

**Affiliations:** 1 Service de Néphrologie-Dialyse, Hôpital Al farabi, Université Mohamed Premier, Faculté de médecine et de pharmacie, Oujda, Maroc; 2 Service d’Orthopédie, Hôpital Al farabi, Université Mohamed Premier, Faculté de médecine et de pharmacie, Oujda, Maroc

**Keywords:** Hémodialyse chronique, troubles phosphocalciques, K/DOQI, KDIGO, chronic hemodialysis, phosphocalcic disorders, K/DOQI, KDIGO

## Abstract

Les troubles phosphocalciques sont fréquents en hémodialyse chronique. Leurs conséquences justifient une prévention et un traitement adaptés aux recommandations des sociétés savantes. L’objectif de notre étude était de déterminer le statut phosphocalcique de nos patients hémodialysés chroniques (HDC) et l’évaluation des taux de conformité des indicateurs aux recommandations K/DOQI et KDIGO. Ainsi, nous avons réalisé une étude transversale incluant les 83 patients HDC du centre d’hémodialyse de l’hôpital Al Farabi d’Oujda. L’âge moyen de nos patients était de 49.8± 15.6 ans. Une prédominance masculine a été notée. La conformité des indicateurs du bilan phosphocalcique chez nos patients hémodialysés chroniques par rapport aux recommandations KDIGO était de l’ordre de 21.6%. Le pourcentage des patients ayant des données phosphocalciques conformes aux cibles recommandées par les K/DOQI était Les patients répondants simultanément aux quatres critères recommandés par les K/DOQI n’étaient que 8.4%.

## Introduction

En hémodialyse chronique (HDC), les troubles du métabolisme minéral et osseux (TMO) sont associés à une morbimortalité élevée [1]. Ces troubles débutent précocement au cours de l’insuffisance rénale chronique (IRC), et justifient une prévention et un traitement adaptés aux recommandations des sociétés savantes [2]. Les kidney disease: improving global outcome (KDIGO) ont élaboré de nouvelles recommandations de bonnes pratiques cliniques dans ce domaine afin de mettre à jour les recommandations américaines kidney disease outcomes quality initiative (KDOQI) publiées en 2003, et de guider les néphrologues dans la prise en charge de ces TMO [3–5]. L’objectif de notre étude était de déterminer le statut phospho-calcique et la prévalence des TMO chez nos hémodialysés chroniques ainsi que le taux de conformité de ces indicateurs aux recommandations K/DOQI et KDIGO.

### Méthodes

Nous avons réalisé une étude transversale, de Octobre 2011 et Janvier 2012 incluant 83 patients hémodialysés chronique au centre d’hémodialyse de l’hôpital Al Farabi d’Oujda, dans l’Oriental Marocain. Nous avons recueilli les données démographiques, cliniques, biologiques, radiologiques et thérapeutiques de nos patients. Enfin, nous avons comparé nos données aux cibles des recommandations:

K/DOQI 2005: la calcémie entre 84 et 95mg/l (2.1-2.35 mmol/l), la phosphorémie entre 35 et 55mg/l (1.13-1.78mmol/l), le produit phosphocalcique < 5500mg^2^/l^2^(<4.51mmol^2^/l^2^), la parathormone intacte entre 150 et 300pg/l (16.5-33 mmol/l).

Les recommandations KDIGO 2009: la calcémie entre 84 et 104 mg/l (2.1-2.6 mmol/l), la phosphorémie entre 32 et 60 mg/l (0.8-1.5mmol/l), la parathormone entre 134 et 603 pg/ml (14.7-66mmol/l).

## Résultats

**Figure 1:**
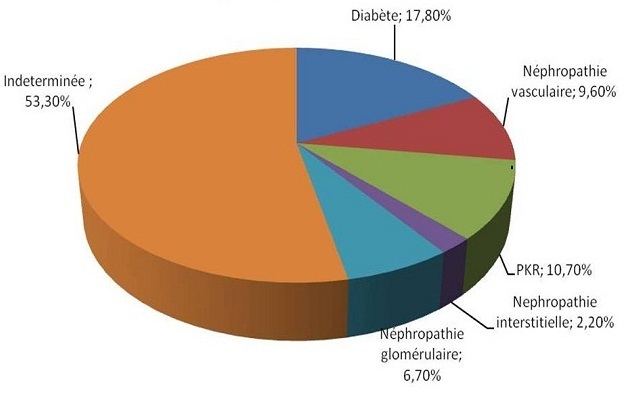
Néphropathies causales chez nos patients hémodialysés chroniques

L’âge moyen de nos patients était de 49.8 ± 15.6 ans. Une prédominance masculine a été notée avec un sexe ratio H/F: 42/38). La durée moyenne d’hémodialyse était de 89± 54 mois. 67(80%) de nos patients étaient de bas niveau socio-économique. La néphropathie causale était diabétique dans 17.8% des cas, vasculaire dans 9.6% des cas, et indéterminée dans 53.3% (Figure 1). Les douleurs osseuses étaient présentes chez 67.4% des patients. 15.6% de nos hémodialysés chroniques souffraient de prurit, et les fractures pathologiques étaient observées chez 7.2% de l’ensemble des patients.

Sur le plan biologique, 56.7% des patients avaient une hyperparathyroidie secondaire avec une PTH 1-84 moyenne de 508+-380 pg/ml. La calcémie moyenne est de 2.06±0.4 mmol/l et la phosphrémoie moyenne est de 1.16±;0.44 mmol/l. La moyenne de la 25 OH vitamine D est de 87±18 nmol/l (Tableau 1). Les radiographies standards du squelette avaient montré des traits de fractures dans 7.5% des cas, des déminéralisations osseuses dans 57% des cas et des calcifications vasculaires dans 19% des cas. 16% des patients présentaient des nodules parathyroïdiens, objectivés à l’échographie cervicale. L’échographie transthoracique a retrouvé 9% de calcifications vasculaires (Tableau 2).

**Tableau 1:** Données biologiques phosphocalciques chez nos patients

Paramètres phosphocalciques	Moyenne± Ecart-type
La parathormone intacte 1-84 (PTHi)pg/ml	508±380
Calcémie (mmol/l)	2,06±0,4
Phosphoremie (mmol/ml)	1,16±0,44
Produit phosphocalcique (mmol^2^/l^2^)	2,39±0,17
25 OH vit D (nmol/l)	87±18
Phosphatase alcaline (PAL) (UI/I)	321,4±173,6

**Tableau 2:** Caractéristiques radiologiques de nos patients hémodialysés chroniques

Caractéristiques radiologiques	N(%)
Nombre de fractures	6(7,5%)
Déminéralisations osseuses	45(57%)
Calcifications vasculaires	15(19%)
Echographie cervicale	13(16%)
Nodules parathyroïdiens	6(9%)

Sur le plan thérapeutique, l’alfacalcidiol était prescrite chez 40.9% des patients à une dose moyenne de 2.45ug/semaine, la vitamine D native était prescrite chez 3% des patients à la dose de 200 000UI/mois.

Les traitements chélateurs de phosphore était à base de carbonate de calcium dans 78.8% des cas à une dose moyenne de 1.04g/j, à base de Sevelamer dans 4.8% des cas à la dose de 2.4g/j, et à base de carbonate de lanthane dans1.5% des cas à la dose de 1.5g/j. Par ailleurs, une parathyroïdectomie 7/8^ème^ était réalisée chez 4.5% de nos patients.

La conformité aux recommandations K/DOQI et KDIGO: La conformité des indicateurs du bilan phosphocalcique chez nos patients hémodialysés chroniques par rapport aux recommandations KDIGO est de l’ordre de 71.2% pour la calcémie, 61% pour la phosphorémie, 43.1% pour la PTH1-84. Sachant que 29.2% des patients avaient une PTH 1-84 >603pg/ml et une PTH 1-84 < 134 pg/ml était notée dans 27.7% des cas. Dix-huit de nos patients hémodialysés chroniques (21.6%) répondaient aux recommandations des KDIGO. Le pourcentage des patients ayant des données phosphocalciques conformes aux cibles recommandées par les K/DOQI était de 42.2%, 46.9%, 78.1% et 20.3% des cas respectivement pour la calcémie, la phosphorémie, le produit phospho-calcique (Ca*Ph), et la PTH1-84. Cinquante et un pour cent des patients avaient un taux de PTH 1-84 supérieur à 300pg/ml et 12.1% de nos hémodialysés chroniques avaient une PTH inférieure à 150pg/ml.

Les patients répondants simultanément aux quatres critères recommandés par les K/DOQI n’étaient que 7(8.4%). A noter que tous les patients répondants aux recommandations K/DOQI répondaient également aux cibles KDIGO.

## Discussion

Dans notre étude, les patients répondants simultanément aux quatres critères recommandés par les K/DOKI n’étaient que 7 patients (9%), ceci rejoint les données de nombreuses études où la plupart des patients n’atteignent pas les cibles des recommandations sus-cités [6–9]. A l’inverse, 18 patients (22.5%) répondaient aux recommandations des KDIGO. Ainsi, dans notre série, on note un taux plus élevé de patients qui répondaient aux critères des KDIGO que ceux des KDOQI, Celà, peut-être expliqué en grande partie par le fait que les nouvelles cibles de la calcémie et de la PTH ont été élargies et semblent plus facilement atteignables. Le pourcentage des patients ayant un bilan phosphocalcique conforme aux cibles recommandées par les K/DOQI était comparable aux données des séries publiées [6-7;9].

Par ailleurs, la conformité des indicateurs du bilan phosphocalcique chez nos patients aux recommandations KDIGO, malgré sa supériorité aux résultats observés avec les KDOQI, demeure insatisfaisante. Ce-ci s’explique aisément par le faible niveau socio-économique de nos patients, dépourvus de couverture sociale dans la grande majorité des cas; rendant une prise en charge thérapeutique à base de chélateurs non calciques de phosphore et/ou des calcimimétiques souvent difficile voire même inconcevable compte tenu de leur coût élevé.

## Conclusion

La prise en charge des TMO chez le patient hémodialysé chronique reste un domaine complexe et en constante évolution. Les dernières recommandations permettent d’appliquer une stratégie thérapeutique individualisée afin d’optimiser la prise en charge de ces troubles. Toutefois, dans un contexte socio-économique délicat, comme le notre, les cibles recommandées par les KDIGO sont plus accessibles que celles des KDOQI. Il va sans dire que le pourcentage de patients qui obéissent à tous les critères demeure non satisfaisants.
